# Virulence and genotypic characterization of *Listeria monocytogenes* isolated from vegetable and soil samples

**DOI:** 10.1186/s12866-014-0241-3

**Published:** 2014-09-08

**Authors:** Dharmendra Kumar Soni, Major Singh, Durg Vijai Singh, Suresh Kumar Dubey

**Affiliations:** Department of Botany, Banaras Hindu University, Varanasi, 221005 India; Indian Institute of Vegetable Research, Varanasi, 221305 India; Infectious Disease Biology, Institute of Life Sciences, Bhubaneswar, 751023 India

**Keywords:** Serotype identification, ERIC- and REP-PCR, Virulence genes, Multiplex PCR

## Abstract

**Background:**

*Listeria monocytogenes,* a foodborne pathogen is ubiquitous to different environments including the agroecosystem. The organism poses serious public health problem. Therefore, an attempt has been made to gain further insight to their antibiotic susceptibility, serotypes and the virulence genes.

**Results:**

Out of the 10 vegetables selected, 6 (brinjal, cauliflower, dolichos-bean, tomato, chappan-kaddu and chilli), 20 isolates (10%) tested positive for *L. monocytogenes*. The prevalence of the pathogen in the respective rhizosphere soil samples was 5%. Noticeably, *L. monocytogenes* was absent from only cabbage, broccoli, palak and cowpea, and also the respective rhizospheric soils. The 30 isolates + ve for pathogenicity, belonged to serogroup 4b, 4d or 4e, and all were positive for *inlA*, *inlC, inlJ*, *plcA*, *prfA, actA*, *hlyA* and *iap* gene except one (VC3) among the vegetable isolates that lacked the *plcA* gene. ERIC- and REP-PCR collectively revealed that isolates from vegetables and their respective rhizospheric soils had distinct PCR fingerprints.

**Conclusions:**

The study demonstrates the prevalence of pathogenic *L. monocytogenes* in the selected agricultural farm samples. The increase in the number of strains resistant to ciprofloxacin and/or cefoxitin seems to pose serious public health consequences.

## Background

*Listeria monocytogenes,* the foodborne pathogen, causes listeriosis with high mortality rates (~30%), and presently considered to pose serious public health problem [1]. The organism survives diverse conditions such as low temperature, low pH and high salt concentrations, and manifests abortion, stillbirth, septicemia, meningitis and meningoencephalitis in pregnant women, neonates, elderly, or immune-compromised humans [2,3]. Usually, *L. monocytogenes* is susceptible to a wide range of antibiotics, but resistance to multiple antibiotics is also on record [4,5]. The presence of a number of virulence factors such as internalins (encoded by *inlA, inlC, inlJ*), listeriolysin O (LLO encoded by *hlyA*), actin (*actA*), phosphatidyl-inositol-phospholipase C (PI-PLC encoded by *plcA*), iap (invasion associated protein encoded by *iap*) and virulence regulator (encoded by *prfA*), in *L. monocytogenes* significantly regulates the pathogenicity [6,7]. Serotyping of *L. monocytogenes* from different sources revealed difference in their virulence attribute [8-10]. The isolates from food and environmental samples belonged to a small number of serotypes 1/2a, 1/2b and 4b [11,12]. Among the various approaches for molecular typing of *L. monocytogenes*, Pulsed Field Gel Electrophoresis (PFGE) has been considered the “gold standard” owing to its high reproducibility and discriminatory ability [13]. However, Repetitive Element Sequence (REP) and Enterobacterial Repetitive Intergenic Consensus (ERIC) - PCR are relatively simple, cost-effective and discriminatory to the type genus *Listeria* which generate DNA fingerprint comparable to PFGE that permits discrimination within a single bacterial species [12,14].

*L. monocytogenes* common to different environments including the agroecosystem, may serve as the contamination source. Since the first report of human listeriosis outbreak in 1980 through consumption of contaminated food [15], several such cases have been reported following the consumption of raw and cooked meat, dairy products and ready-to-eat foods, and raw and smoked seafood [16]. Todd and Notermans, [17] and Swaminathan and Gerner-Smidt [18] reported outbreaks of foodborne listeriosis across the various countries. The listeriosis incidence varies from 0.3 to 11.3 per million population in different countries, although no such outbreaks have been reported from India [19]. The incidence of listeriosis has also been attributed to consumption of salad vegetables such as cabbage, celery, lettuce, cucumber, onion, leeks, watercress, radish, tomatoes, and fennel [20,21]. Since the majority of such studies used vegetable samples randomly collected from the market, it is difficult to account for the actual inoculum source.

India is the second largest global producer of the fruits (45.5 million tones/y) and vegetables (90.8 million tones/y), contributing 10.23% and 14.45% of the total world production, respectively [22]. Therefore, it is essential to continuously monitor the prevalence of foodborne pathogens including *L. monocytogenes.* A few Indian reports available, show prevalence of *L. monocytogenes* in different vegetable and soil samples, but these are limited to the virulence attributes, antibiotics sensitivity and sub-typing of the isolates [23-25]. The organism poses serious problem to the food industry, public health agencies, and government bodies [9,26]. In the present study, we characterized *L. monocytogenes* isolated from the vegetables and the respective rhizosphere soils (soil adhering to the root surface) for the presence of virulence genes, serovar and antibiotics susceptibility. For generation of DNA fingerprints and to know-how of the clonal relationships among the isolates, ERIC- and REP-PCR approach was used.

## Results and discussion

### Prevalence of *L. monocytogenes*

The overall prevalence of *L. monocytogenes* in 200 vegetable samples was 20 (10%) and 10 (5%) for 200 soil samples. Of the 10 vegetables, 6 of these i.e., brinjal, cauliflower, dolichos-bean, tomato, chappan-kaddu and chilli and their respective rhizospheric soils tested + ve for *L. monocytogenes*. Conversely, cabbage, broccoli, palak and cowpea and their respective soils tested - ve.

Reports elsewhere in other countries indicated variable prevalence of *L. monocytogenes* in vegetables. It was low (0.62%) in North China, 3.1% in Brazil and high (60%) in US [26-28]. The findings of the present study on the prevalence of *L. monocytogenes* in vegetable samples, are in agreement with the values (10% and 11%) from the freshly supermarket prepared, cooked or raw ready-to-eat vegetable-salads from Santiago, Chile and Japanese light pickle made from vegetables in Obihiro, respectively [20,29]. Studies conducted on vegetable samples in Mumbai and Tamilnadu in India reported an average 13% prevalence of *L. monocytogenes* [23,25].

Among the 10 vegetables, *L. monocytogenes* frequency was 20% (4 contaminated samples out of 20 analyzed) in brinjal, cauliflower, chappan-kaddu and chilly while 10% (2 contaminated samples out of 20 analyzed) in dolichos-beans and tomato. *L. monocytogenes* has previously been isolated from carrot, cabbage, tomato, cucumber, green beans, broccoli, spinach etc. [20,21,27]. In India, *L. monocytogenes* is reported from coriander leaves (50%), tomato (11%), cabbage (25%), spinach (50%) and *Brassica oleracea* (100%) [23,25].

Further the overall prevalence of *L. monocytogenes* in the rhizospheric soil samples from agricultural farm, is in accordance with the value (5%) as reported by Moshtaghi *et al.,* [24] from Hisar, India and also, the *L. monocytogenes* prevalence (5.3%) from soils of calf-cow operation, California, U.S. [30]. However, a higher prevalence of *L. monocytogenes* (100% and 23%) is reported in soils from animal farm environment in Nsukka, Nigeria and New York, U.S., respectively [31,32]. In soils from the respective vegetables, *L. monocytogenes* prevalence reached 10% (2 samples contaminated out of 20) in case of cauliflower, chappan-kaddu, chilly and dolichos-beans, while 5% (1 sample out of 20) in the rhizospheric soil of brinjal and tomato.

In the present study, vegetables such as cabbage, broccoli, palak and cowpea and their respective rhizospheric soils tested – ve for *L. monocytogenes*. Although, previous studies in India and elsewhere observed the presence *L. monocytogenes* in such vegetable samples [20,25,27,33]. In the current study, the apparent variation in the association of *L. monocytogenes* with the selected vegetables or the rhizospheric soils, possibly reflects the consequences of a sort of crop- or soil- specific interaction with its pathogen. However, Garrec *et al.,* [34] and Vivant *et al.,* [35] observed low pathogen population in a heavily contaminated environment thus limiting its isolation or even the microbes. Although a few studies showed carrot to have anti-listerial activity, while cabbage highly inhibitory to Gram – ve microorganisms along with some suppressant effect against Gram + ve ones such as *L. monocytogenes* [36]*.* The proliferation of *L. monocytogenes* in the vegetable samples depends upon several factors and their complex interactions like intrinsic properties of the food (e.g. pH, NaCl content, water activity, composition, associated microflora, antimicrobial constituents), extrinsic factors (e.g. temperature, gas atmosphere), the physico-chemical environment of the plant surface, epiphytic fitness, biofilm formation, and bacteria–bacteria and vegetable-bacteria interactions [36,37]. Soil is the environmental niche of *L. monocytogenes* but its composition, microbial communities and macrofauna, are the extrinsic edaphic factors that regulate the fate of *L. monocytogenes* in the soil. Generally, suppression of microflora via soil sterilization permitted better growth of *L. monocytogenes* than the competitive microflora [38]. As such, deciphering environmental drivers that impact the occurrence of *L. monocytogenes* in soils is extremely hard as these are interconnected, and extrinsic factors (edaphic parameters, biotic environment, etc.) affect their survival [35,38]. Therefore, understanding the condition that triggers contamination or, on conversely that limits risks of contamination, is difficult in face of the complexity of the ecology of *Listeria*.

### Antibiotics susceptibility

*L. monocytogenes* isolates from vegetable and their rhizospheric soil samples were tested for their antibiotic susceptibility. Out of 20 isolates from vegetable samples, 15 were resistant to ciprofloxacin and cefoxitin, while 3 only to ciprofloxacin, and 2 to cefoxitin. Similarly, out of 10 isolates from soil samples, 5 were resistant to ciprofloxacin and cefoxitin while only 4 to cefoxitin, and only 1 resistant to ciprofloxacin. All the isolates, however, were susceptible to other antibiotics tested (Table 1).

**Table 1:** **Source of isolation, serogroup, antibiogram, ERIC- and REP- fingerprints and virulence profiles of** ***L. monocytogenes*** **used in this study**

**Sl. no.**	**Strains**	**Source of isolation**	**Date of isolation**	**Serogroup**	**Antibiogram**	**ERIC type**	**REP type**	**Presence of following genes determined by PCR**							
								***inlA***	***inlC***	***inlJ***	***plcA***	***prfA***	***actA***	***hlyA***	***iap***
1	VB1	Vegetable-brinjal	15.10.2011	4b, 4d, 4e	Cf, Fox	XVIA	XIIIA	+	+	+	+	+	+	+	+
2	VB2	Vegetable-brinjal	15.10.2011	4b, 4d, 4e	Cf, Fox	XVIA	XIIIA	+	+	+	+	+	+	+	+
3	VB3	Vegetable-brinjal	15.10.2011	4b, 4d, 4e	Cf, Fox	XVIA	XIIIA	+	+	+	+	+	+	+	+
4	VB4	Vegetable-brinjal	15.10.2011	4b, 4d, 4e	Cf, Fox	XVIA	XIIIA	+	+	+	+	+	+	+	+
5	VCF1	Vegetable-cauliflower	15.11.2011	4b, 4d, 4e	Cf, Fox	XVIB	XIIIA	+	+	+	+	+	+	+	+
6	VCF2	Vegetable-cauliflower	15.11.2011	4b, 4d, 4e	Cf, Fox	XVIC	XIIIB	+	+	+	+	+	+	+	+
7	VCF3	Vegetable-cauliflower	15.11.2011	4b, 4d, 4e	Cf, Fox	XVIIB	XIIIB	+	+	+	+	+	+	+	+
8	VCF4	Vegetable-cauliflower	15.11.2011	4b, 4d, 4e	Cf, Fox	XVIIB	XIIIB	+	+	+	+	+	+	+	+
9	VDB1	Vegetable-dolichos bean	15.12.2011	4b, 4d, 4e	Cf	XVIIA	XIIIB	+	+	+	+	+	+	+	+
10	VDB2	Vegetable-dolichos bean	15.12.2011	4b, 4d, 4e	Cf, Fox	XVIIA	XIIIB	+	+	+	+	+	+	+	+
11	VT1	Vegetable-tomato	15.01.2012	4b, 4d, 4e	Cf, Fox	XVB	XIVB	+	+	+	+	+	+	+	+
12	VT2	Vegetable-tomato	15.01.2012	4b, 4d, 4e	Cf, Fox	XVA	XIVB	+	+	+	+	+	+	+	+
13	VCK1	Vegetable-chappan kaddu	15.01.2012	4b, 4d, 4e	Cf, Fox	XVA	XIVC	+	+	+	+	+	+	+	+
14	VCK2	Vegetable-chappan kaddu	15.01.2012	4b, 4d, 4e	Cf	XVC	XIVB	+	+	+	+	+	+	+	+
15	VCK3	Vegetable-chappan kaddu	15.01.2012	4b, 4d, 4e	Cf, Fox	XIV	XIVB	+	+	+	+	+	+	+	+
16	VCK4	Vegetable-chappan kaddu	15.01.2012	4b, 4d, 4e	Cf	XIV	XIVD	+	+	+	+	+	+	+	+
17	VC1	Vegetable-chilli	15.02.2012	4b, 4d, 4e	Fox	XIII	XIVB	+	+	+	+	+	+	+	+
18	VC2	Vegetable-chilli	15.02.2012	4b, 4d, 4e	Cf, Fox	XIII	XIVA	+	+	+	+	+	+	+	+
19	VC3	Vegetable-chilli	15.02.2012	4b, 4d, 4e	Fox	XIII	XIVA	+	+	+	-	+	+	+	+
20	VC4	Vegetable-chilli	15.02.2012	4b, 4d, 4e	Cf, Fox	XIII	XIVA	+	+	+	+	+	+	+	+
21	S1	Soil from brinjal field	15.10.2011	4b, 4d, 4e	Cf, Fox	XIXA	XVA	+	+	+	+	+	+	+	+
22	S2	Soil from cauliflower field	15.11.2011	4b, 4d, 4e	Cf, Fox	XIXA	XVA	+	+	+	+	+	+	+	+
23	S3	Soil from cauliflower field	15.11.2011	4b, 4d, 4e	Fox	XIXA	XVA	+	+	+	+	+	+	+	+
24	S4	Soil from dolichos bean field	15.12.2011	4b, 4d, 4e	Cf	XIXB	XVA	+	+	+	+	+	+	+	+
25	S5	Soil from dolichos bean field	15.12.2011	4b, 4d, 4e	Cf, Fox	XX	XVA	+	+	+	+	+	+	+	+
26	S6	Soil from tomato field	15.01.2012	4b, 4d, 4e	Fox	XVIIIB	XVIII	+	+	+	+	+	+	+	+
27	S7	Soil from chappan kaddu field	15.01.2012	4b, 4d, 4e	Fox	XVIIIA	XVII	+	+	+	+	+	+	+	+
28	S8	Soil from chappan kaddu field	15.01.2012	4b, 4d, 4e	Cf, Fox	XVIIIA	XVI	+	+	+	+	+	+	+	+
29	S9	Soil from chilli field	15.02.2012	4b, 4d, 4e	Fox	XVIIIC	XVII	+	+	+	+	+	+	+	+
30	S10	Soil from chilli field	15.02.2012	4b, 4d, 4e	Cf, Fox	XVIIIC	XVII	+	+	+	+	+	+	+	+

Cf: ciprofloxacin; Fox: cefoxitin.

The multidrug-resistant *L. monocytogenes* associated with human listeriosis, has been reported from food and environment [39]. Moreover, *L. monocytogenes* resistant to ampicillin, erythromycin, gentamicin, kanamycin, penicillin, streptomycin, sulphonamide, trimethoprim, tetracycline, and rifampicin has also been documented [4,26]. In India, Dhanashree *et al.,* [33] reported sensitivity of *L. monocytogenes* to ampicillin, ciprofloxacin, cotrimoxazole, erythromycin, penicillin and chloramphenicol. Sharma *et al.,* [40] and Soni *et al.,* [41] isolated multi-drug resistant *L. monocytogenes* from human clinical samples, water and milk. The application of commonly used antibiotics in humans and veterinary, the disposal of untreated effluents in the environment, and the application of faeces or dung slurries of infected (or carrier) animals onto the agricultural land, have role(s) in the development of resistance in the pathogens [31,42]. There is also the possibility of the spread of multidrug-resistant bacteria through intake of uncooked food, and may have severe medical and public health implications [5,43]. The resistance of all the isolates from soil and vegetables to ciprofloxacin and/or cefoxitin as observed in this study, indicates the possible emergence of antibiotic resistance in *L. monocytogenes*. This finding is significant in context of the incidence of temporal and spatial changes in the antibiotics resistance [26,44]. Therefore, there is a need for the continuous surveillance of the emergence of antibiotics resistance.

### Species and serovar identification

Twenty isolates from vegetable samples were + ve for internalin A (*inlA*) gene indicating that all of them belonged to *L. monocytogenes*, and in serotype specific multiplex PCR, all the isolates were + ve for ORF2110 and ORF2819 gene indicating that these belonged to 4b, 4d or 4e serogroup. Similarly, 10 isolates from the soil were also + ve for internalin A (*inlA*) gene, and the serotyping showed them + ve for ORF2110 and ORF2819 (Table 1).

The typing of *L. monocytogenes* is important in epidemiological studies due to the relationship between serotypes and food-borne listeriosis, and to identify the source of contamination and the dissemination routes. As serotypes 4d and 4e are relatively rare in food, the isolates belonging to 4b, 4d or 4e serogroup may be regarded as serotype 4b [9,45]. The present observations thus corroborate with those of others on the isolation of serogroup 4b from vegetable samples [27,29]. This study also shows the high prevalence of 4b serotype among the *L. monocytogenes*, that is commonly associated with human listeriosis. The high incidence of this serotype in vegetable and soils may be of serious concern from the food safety perspective view point.

### Virulence associated genes

Twenty isolates of *L. monocytogenes* from vegetable and 10 from soil samples were screened for the presence of virulence genes. All the 20 isolates from vegetable tested + ve for *inlA*, *inlC, inlJ*, *plcA*, *prfA, actA*, *hlyA* and *iap* genes except 1 (VC3) that lacked *plcA*. Similarly, all the 10 isolates from soil were + ve for *inlA*, *inlC, inlJ*, *plcA*, *prfA, actA*, *hlyA* and *iap* gene (Table 1).

*L. monocytogenes* strains vary in their virulence potential. Whereas some of the *L. monocytogenes* strains are naturally virulent to inflict high morbidity and mortality, others non-virulent and unable to infect the mammalian host [46,47]. The discrimination between pathogenic and non-pathogenic strains is imperative to assess the possible significance of this microorganism from food safety and public health aspects [48,49]. Several protocols developed for the assessment of *L. monocytogenes* virulence include *in vivo* bioassay and *in vitro* cell assay. The *in vivo* method has limitations because of its expensive nature, and the use of animals. The *in vitro* assay is hampered by the lack of desired reproducibility and the time consumed during analysis. PCR based assays for the key virulence-associated genes yield rapid and reproducible results. Few studies based on the presence of key virulence proteins and genes in the whole spectrum of *L. monocytogenes* strains have contributed to the acceptable outcome [50]. This study also demonstrates that all the *L. monocytogenes* isolates, irrespective of their source, possessed internalin *inlC* and *inlJ* genes indicating ability for their cellular internalization. Majority of the isolates from vegetables and soil possessed virulence genes encoding *inlA, inlC, inlJ, plcA, prfA, actA, hlyA* and *iap,* indicating that these possess all the requisites of a virulent strain. These findings are similar to the isolation of virulent *L. monocytogenes* from vegetable and soil as reported by Chen *et al.,* [12], Maklon *et al.,* [29] and Sant Ana *et al.,* [27]. Moreover, one of the isolates from vegetables (VC3) lacking *plcA* showed variation in the virulence gene profile, and this could be because of the absence of the respective virulence gene or the incidence of some mutations in the same gene [43,51].

### ERIC- and REP-PCR fingerprint analysis

**Figure 1:**
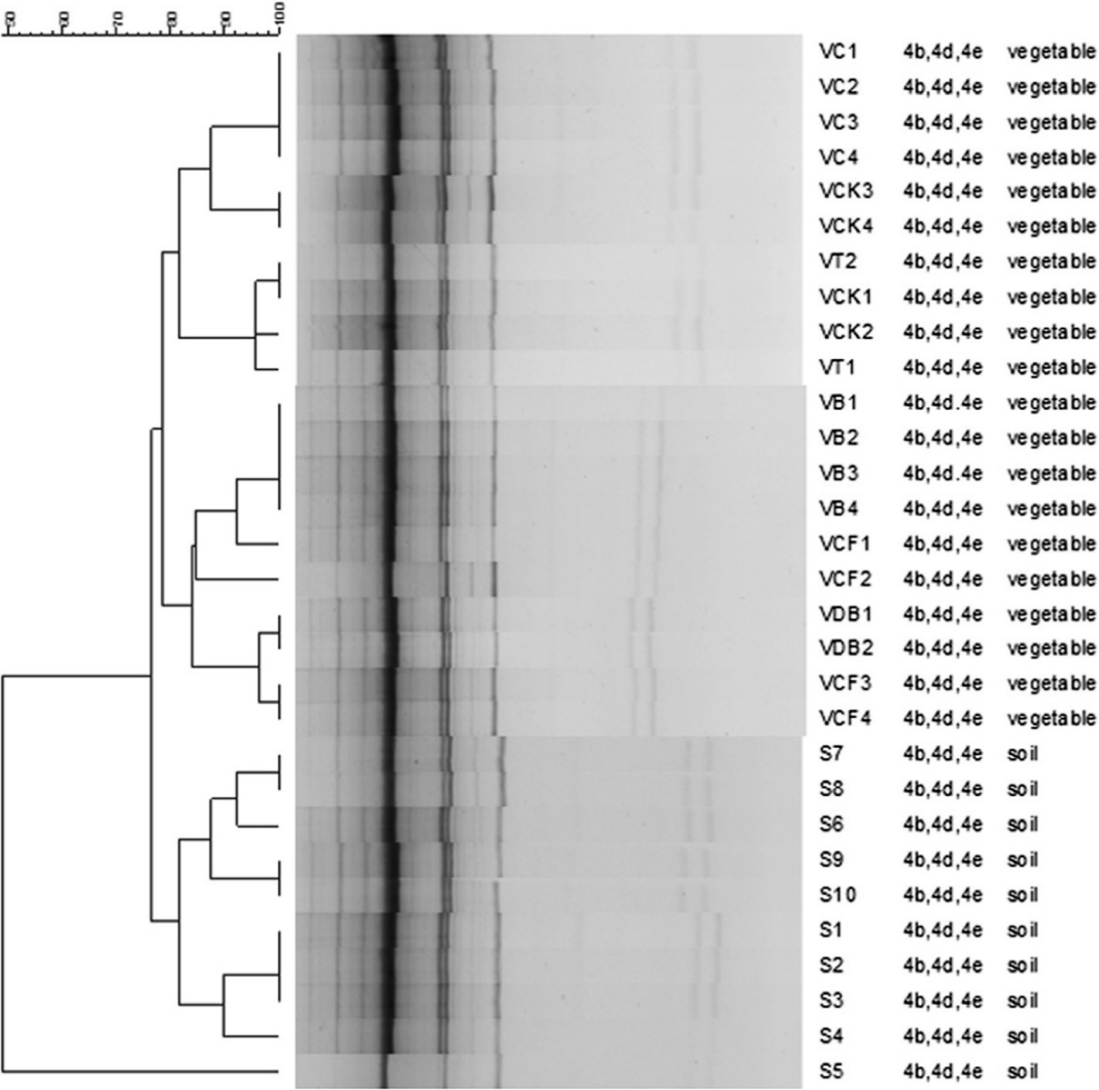
**DNA fingerprints generated by ERIC-PCR amplification from vegetable and soil isolates of** ***L. monocytogenes*** **.** The dendrogram was generated using the Bionumerics Fingerprint Analyst Software (Applied Maths), and data clustered by the unweighted pair group method with arithmetic means. Similarity of the ERIC-PCR fingerprint profiles was calculated using the average simple-match similarity matrix and the default cluster settings of 0.00% optimization and 1.00% band position tolerance were used.

The ERIC-PCR of genomic DNA from *L. monocytogenes* isolates from soil and vegetables yielded a total of 8 fingerprint profiles (profiles XIII through XIX) not described earlier, and consisted of 7 to 12 bands ranging between 350 and 5200 bases (Figure 1). While 2 of the 10 isolates from the soil, showed identical fingerprint profile, other 3 isolates had almost similar ones. Similarly, 3 isolates also yielded identical profile, and 1 isolate had closely related one. Isolates from vegetables yielded five distinct fingerprints, whereas those from chilli had identical fingerprint, few isolates from chappan-kaddu showed the closely related pattern. Although remaining isolates from chappan-kaddu showed distinct fingerprints, those from tomato had closely related fingerprint patterns. Whereas isolates from brinjal had identical fingerprint profile, those from cauliflower and dolichos-bean were characterized by distinct but closely related fingerprints. There was no relationship among the fingerprint profiles of vegetable and soil isolates.

**Figure 2:**
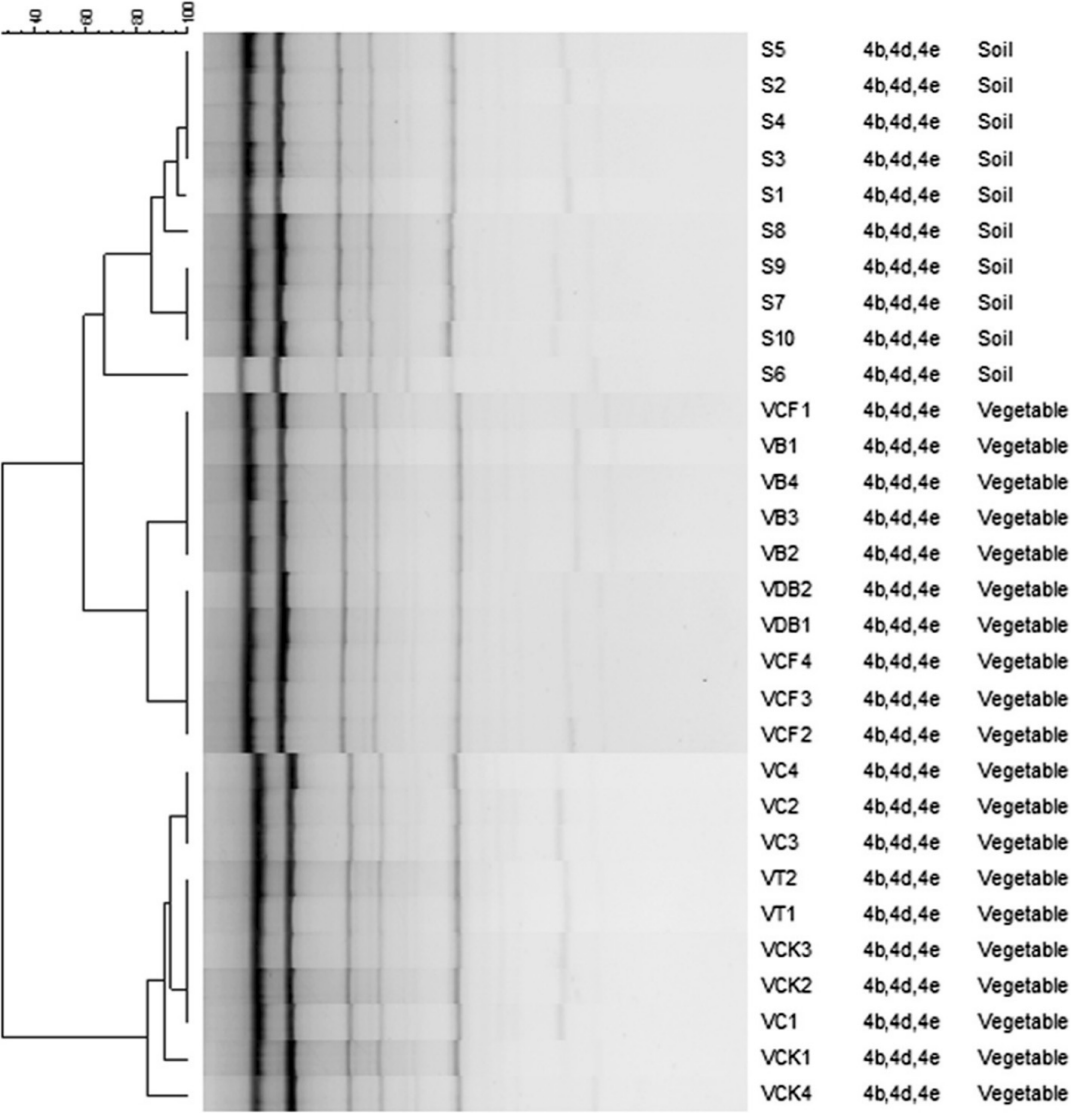
**DNA fingerprints generated by REP-PCR amplification from vegetable and soil isolates of** ***L. monocytogenes*** **.** The dendrogram was constructed using the Bionumerics Fingerprint Analyst Software (Applied Maths) as described in the legend of Figure 1.

The REP-PCR of genomic DNA from *L. monocytogenes* from soil and vegetables showed amplification of multiple DNA fragments (450 to 6000 base) (Figure 2). Likewise, the ERIC-profile of 6 isolates from soil revealed identical to closely related fingerprints and while the remaining isolates had related to distinct fingerprints. Whereas isolates from brinjal, cauliflower and dolichos-bean had similar to identical fingerprints, isolates from chilli, tomato or chappan-kaddu had identical but different fingerprints. No correlation in fingerprint profile between the soil and vegetable isolates was observed.

ERIC- sequences located in the extragenic regions of the bacterial genome are 124 to 127 bases long elements consisting of highly conserved central inverted repeat. REP elements containing 6 degenerate positions, are 38-bp long with a 5-bp variable loop between each side of the conserved palindrome [14,52,53]. ERIC- and REP-PCR was used in this study to assess the relatedness of *L. monocytogenes* from the soil and vegetable, and of the serotypes. Soil isolates showed identical to similar and closely related ERIC- and REP fingerprints but distinct from the vegetable isolates indicating distinct association of strains in soils and vegetables. It is possible that many clonal types of the organism populate the soil but only distinct clones of *L. monocytogenes* colonize the plants. The failure to isolate strains from the roots of some plants may be due to hyperproduction of antagonists by the roots or the associated antagonist i.e., microflora. Several workers reported recovery of isolates from vegetables originating from the soil and/or the environment used for their farming [15,21,54]. In the overall, there is a strong correlation of the results obtained from the PCR, and the isolates from soils yielded identical fingerprint but distinct from the vegetable counterparts. The observed difference in the banding pattern among the isolates from soil and vegetables suggests the possible divergence in the genomic organization arising from the genetic re-assortment in the given ecological niche over time. There was no correlation between serogroup and the PCR fingerprint profiles.

## Conclusions

In conclusion, *L. monocytogenes* isolates recovered from vegetable and soil samples belonging to 4b, 4d or 4e serogroups, exhibited multiple antibiotic resistances and the presence of all the virulence genes. The study provides evidence for the prevalence of pathogen i.e., *L. monocytogenes* in farm samples though there is no clear-cut evidence of listeriosis outbreak in India. In addition, the acquisition of antibiotic resistance in the isolates studied, reflects the potential public health consequences.

## Methods

### Study site and sample collection

A total of 400 samples were collected from the agricultural farm of the Indian Institute of Vegetable Research (IIVR), Varanasi, India (25° 08’ N latitude; 83° 03’ E longitude and 90 m from sea level), through October 2011 to February 2012, and of which, 200 each were from vegetables and the accompanying rhizospheric soils. Among the vegetables, 20 each were from brinjal (*Solanum melongena*), cabbage (*Brassica oleracea* var*. capitata*), broccoli (*Brassica oleracea* var*. italica*), cauliflower (*Brassica oleracea* var*. botrytis*), dolichos-bean (*Dolichos lablab*), palak (*Beta vulgaris*), tomato (*Solanum lycopersicum*), chappan-kaddu (*Cucurbita pepo*), chilli (*Capsicum annum*) and cowpea (*Vigna unguiculata*).

Rhizosphere soil (200 samples) contained 20 each from the same vegetable grown. Soil samples were collected at the ripening stage by uprooting the plants. Roots were vigorously shaken to separate the loosely bound bulk soil. Pooled soil samples from the vegetable field were homogeneously mixed and sieved (2 mm) to remove the plant debris prior to further analysis [55]. All the vegetable and rhizospheric soil samples were collected aseptically, transported chilled to the laboratory, and processed within 24 h of collection.

### Isolation and identification of *L. monocytogenes*

Vegetable and soil samples were examined following the standard double enrichment method as prescribed by ISO 11290:1 with slight modifications [56]. Each vegetable sample was cut into small pieces, then 25 g each of vegetable and soil sample were separately placed in Stomacher bag with 225 ml of half-Fraser broth (Difco, USA), and homogenized using Stomacher (60 s). The bag was incubated (24 h, 30°C). Second enrichment was done by adding 0.1 ml from the overnight grown culture into 10 ml of the full strength of the selective agents (Fraser broth, Difco, USA), and incubated (48 h, 37°C) with the subsequent spreading on PALCAM agar (Difco), and re-incubated (48 h, 37°C). Grey-greenish colonies with black sunken centre and black halo were picked up and confirmed by Gram staining, biochemical tests such as catalase, methyl red-Voges-Proskauer (MR-VP) reaction, nitrate reduction, motility (20-25°C), acid production from rhamnose, xylose, mannitol, α-methyl-D-mannopyranoside, and CAMP test with *Staphylococcus aureus* and *Rhodococcus equi* [57]. *L. monocytogenes* MTCC1143, *S. aureus* MTCC1144 and *R. equi* MTCC1135 served as control. All the *L. monocytogenes* isolates and control strains were preserved in tryptic soy agar slants at room temperature for use in the subsequent analysis.

### Antibiotics susceptibility test

All *L. monocytogenes* isolates were tested for their susceptibility to 10 antibiotics commonly used in veterinary and human therapy, using the disc diffusion method of Bauer *et al.,* [58]. Antibiotics discs (Oxoid, UK) with the following concentrations were used: ampicillin (A, 10 μg), chloramphenicol (C, 30 μg), ciprofloxacin (Cf, 5 μg), cefoxitin (Fox, 30 μg), co-trimoxazole (SXT, 25 μg), gentamicin (G, 10 μg), oflaxacin (Of, 5 μg), rifampicin (R, 5 μg), streptomycin (S, 10 μg), and tetracycline (T, 30 μg). The diameter of the clearance zone was recorded and interpreted following the guidelines of the Clinical and Laboratory Standards Institute (CLSI) for Gram + ve bacteria [59].

### DNA isolation

Chromosomal DNA was extracted from *L. monocytogenes* isolates grown overnight (37°C) with shaking (200 oscillations per min) in brain heart infusion broth (BHIB, Difco, USA) following the protocol of QIAGEN DNeasy® Blood & Tissue kit. Harvested biomass (maximum 2 × 10^9^ cells) were centrifuged (7500 rpm, 10 min), re-suspended in 180 μl lysis buffer [20 mM Tris-Cl (pH 8.0), 2 mM NaEDTA, 1.2% Triton® X-100, 20 mg lysozyme (Sigma) per ml], and incubated for 30 min (37°C). Proteinase K (25 μl) and 200 μl Buffer AL (without ethanol) were added, mixed by vortexing and the mixture re-incubated at 56°C (30 min). Thereafter, 4 μl RNase A (100 mg/ml) was added and incubated (2 min) at room temperature. Pure ethanol (200 μl) was added to the sample, and mixed by vortexing. The DNA was eluted in AE Buffer, and the concentration and purity determined with the help of Eppendorf spectrophotometer at 260 and 280 nm, respectively.

### Species- and virulence- specific genes and serogroup identification

The presence of internalin genes (*inlA, inlC and inlJ)*, virulence-associated genes (*plcA*, *actA*, *hlyA*, *iap* and *prfA)* and serogroup (1/2a, 1/2b, 1/2c, and 4b) was determined by multiplex PCR as described by Liu *et al.,* [7], Notermans *et al.,* [60] and Doumith *et al.,* [8], respectively, and subsequently modified by Soni *et al.,* [41]. The PCR products were analyzed by agarose (1.5%) gel electrophoresis, stained with ethidium bromide, and visualized under UV transilluminator (Bio-Rad). The details of oligonucleotide sequences (Sigma) and PCR cyclic conditions used in this study, are given in Table 2.

**Table 2:** **Sequences and PCR cyclic conditions of primers used for detection of selected serogroups, virulence genes and molecular typing**

**Target gene**	**Primer sequence (5’-3’)**	**Direction**	**Amplicon size(bp)**	**PCR cyclic conditions**	**References**
*lmoO737*	AGG GCT TCA AGG ACT TAC CC	F	691	94°C × 5*'*; (94°C × 30s, 54°C × 75 s, 72°C × 75 s)_35_; 72°C × 10*'*	[8]
	ACG ATT TCT GCT TGC CAT TC	R			
*lmo1118*	AGG GGT CTT AAA TCC TGG AA	F	906	Do	[8]
	CGG CTT GTT CGG CAT ACT TA	R			
ORF2819	AGC AAA ATG CCA AAA CTC GT	F	471	Do	[8]
	CAT CAC TAA AGC CTC CCA TTG	R			
ORF2110	AGT GGA CAA TTG ATT GGT GAA	F	597	Do	[8]
	CAT CCA TCC CTT ACT TTG GAC	R			
*Prs*	GCT GAA GAG ATT GCG AAA GAA G	F	370	Do	[8]
	CAA AGA AAC CTT GGA TTT GCG G	R			
*inlA*	ACG AGT AAC GGG ACA AAT GC	F	800	94°C × 2*'*; (94°C × 20s, 55°C × 20s, 72°C × 50s)_30_; 72°C × 2*'*	[7]
	CCC GAC AGT GGT GCT AGA TT	R			
*inlC*	AAT TCC CAC AGG ACA CAA CC	F	517	Do	[7]
	CGG GAA TGC AAT TTT TCA CTA	R			
*inlJ*	TGT AAC CCC GCT TAC ACA GTT	F	238	Do	[7]
	AGC GGC TTG GCA GTC TAA TA	R			
*plcA*	CTG CTT GAG CGT TCA TGT CTC ATC CCC C	F	1484	95°C × 2*'*; (95°C × 15 s, 60°C × 30s, 72°C × 90s)_35_; 72°C × 10*'*	[60]
	CAT GGG TTT CAC TCT CCT TCT AC	R			
*prfA*	CTG TTG GAG CTC TTC TTG GTG AAG CAA TCG	F	1060	Do	[60]
	AGC AAC CTC GGT ACC ATA TAC TAA CTC	R			
*actA*	CGC CGC GGA AAT TAA AAA AAG A	F	839	Do	[61]
	ACG AAG GAA CCG GGC TGC TAG	R			
*hlyA*	GCA GTT GCA AGC GCT TGG AGT GAA	F	456	Do	[62]
	GCA ACG TAT CCT CCA GAG TGA TCG	R			
*Iap*	ACA AGC TGC ACC TGT TGC AG	F	131	Do	[63]
	TGA CAG CGT GTG TAG TAG CA	R			
*REP1R-I*	IIIICGICGICATCIGGC	F	Several	95°C × 7*'*; (95°C × 1*'*, 44°C × 1*'*, 65°C × 8*'*)_30_; 65°C × 10*'*	[52]
*REP2-I*	ICGICTTATCIGGCCTAC	R			
*ERIC1R*	ATGTAAGCTCCTGGGGATTCAC	F	Several	95°C × 7*'*; (95°C × 1*'*, 52°C × 1*'*, 65°C × 8*'*)_30_; 65°C × 10*'*	[64]
*ERIC2*	AAGTAAGTGACTGGGGTGAGCG	R			

### Genomic fingerprinting by ERIC- and REP-PCR

ERIC- and REP- PCR were performed as described by Rivera *et al.,* [52] and Versalovic *et al.,* [64], respectively. The amplicons were electrophoresed on 1.8% agarose at 60 V (6 h), stained with ethidium bromide and analyzed as described [41]. The fingerprint pattern was measured in a Fluoro-S-Imager (Bio-Rad) and analyzed using Bionumerics fingerprint analyst (Applied Maths, Kortrejik, Belgium) software with a simple-matching similarity matrix, and the data were clustered by the un-weighted pair group method with arithmetic means (UPGMA). The clustering analysis of the ERIC- and REP-PCR patterns could be affected by factors like position bias in gels, band assignment, and different settings in the BioNumerics software. Therefore, the similarity of the ERIC- and REP-PCR fingerprint profiles was calculated using the average simple-match similarity matrix and the default cluster settings of 0% optimization and 1% band position tolerance.
